# Animal Tourism: Thai Caregivers’ Perspectives on Their Relationships with Elephants and Tigers

**DOI:** 10.3390/ani12060790

**Published:** 2022-03-21

**Authors:** Pornpimol Traci Hayward, Serene Liu, Abigail P. Thigpen, Lynette A. Hart

**Affiliations:** 1 Department of Population Health and Reproduction, School of Veterinary Medicine, University of California, Davis, CA 95616, USA; phayward@ucdavis.edu (P.T.H.); apthigpen@ucdavis.edu (A.P.T.); 2 Graduate Group in Epidemiology, University of California, Davis, CA 95616, USA; serliu@ucdavis.edu

**Keywords:** elephant welfare, tiger welfare, human-animal interactions, job satisfaction, protected contact, quality of life, Thailand, tourist industry, wildlife tourism, zoos

## Abstract

**Simple Summary:**

Logging prohibition in Thailand national parks in 1989 ended work for most elephants and mahouts. Animal tourism developed, affording food and husbandry for elephants, and introducing tourism with young tigers. Media and research on wild animals in tourism have explored the animals’ welfare, but not how tourism shifts human-animal relationships and affects the animal caregivers. Caregivers of elephants (*n* = 55) or tigers (*n* = 18) in both private and government tourism facilities in four cities were interviewed in Thai concerning how contexts and management styles impact the relationship between captive animals and caregivers. Mahouts working in private facilities used one-to-one management and were younger and more poorly compensated than those working at government-funded tourism facilities. Tiger caregivers in tourism had direct contact with young tigers, with group management; these caregivers also were younger than in government facilities, with fewer benefits. Most mahouts considered their elephants as family members; a slight majority of these mahouts questioned the ethics of this use of elephants. Tiger caregivers classified tigers as family or friend equally often; one-third of all declined answering whether they approved of use of tigers in tourism. While somewhat solving problems, animal tourism also creates some challenges.

**Abstract:**

This study explored the perspectives of elephant mahouts (*n* = 55) and tiger caregivers (*n* = 18) working in 4 private or 2 government facilities in Thailand to learn their experiences and viewpoints pertaining to use of animals in tourism. Interviews were conducted in Thailand at facilities in four cities. Mahouts working in private tourism facilities used one-to-one management and were significantly younger and more poorly compensated than those working at government-funded zoos, where some had shifted to group management. Tiger caregivers in tourism had direct contact with young tigers, with group management; these caregivers also were significantly younger than in government zoos, and with fewer benefits. Mahouts and tiger caregivers differed in how they viewed their relationships with their animals. Most mahouts considered their elephants as family members; a slight majority of these questioned the ethics of use of elephants in tourism. Tiger caregivers classified tigers as family or friend equally often; one-third of tiger caregivers declined answering on their approval of using tigers in tourism. What to do with aging tigers is a problem; this may explain some tiger caregivers’ reticence to answer questions about using young tigers in tourism. While solving some problems, animal tourism creates several challenges.

## 1. Introduction

People in Thailand commonly experience relationships with a variety of animals, including elephants, which have cultural and religious importance. Changes in government policies and increasing commercialization of animal tourism have led to many captive exotic animals being trained by humans who also provide their care—a type of employment that has expanded with this type of tourism. Animal tourism—specifically, elephant and tiger tourism–has become an important source of income for the Thai government and economy. These unique emerging human-animal relationships that commonly occur in Thailand make it possible to explore associations between the caregivers’ quality of life and their relationships with their animals [1,2].

One of the oldest human-animal relationships is between elephant keepers (mahouts) and their elephants [3,4]. The Beast of Burden Act 1939 has been in use since elephants were still a means of transport in Thailand. Throughout many Asian countries, mahouts traditionally had lifelong special relationships with their elephants, a role often continuing through generations for the men in families [5,6]. Under the elephant legislation section in Thailand pertaining to these elephants domesticated by training, not genetics, elephants are registered as draught animals for doing work and considered as private property. However, one aspect of this relationship changed in Thailand with the modified government legislation in 1989. Logging in Thai National Parks, that had been a traditional activity, was banned [7]. This logging prohibition made illegal the jobs of mahouts for 70% of Thailand’s working elephants. Most mahouts lost their livelihoods and then moved from city to city with their elephants as transient tourist attractions to afford food for their elephants. This activity too was subsequently banned; elephants and their mahouts have since relocated to new jobs in tourism facilities and elephant sanctuaries [8].

Most Thai elephants and their mahouts now work at newer positions in tourism venues, especially in Chiang Mai. Concern about the elephants’ welfare has grown. Research documents that management practices for these elephants sometimes have caused poor body condition, foot problems, and wounding [9]. Feeding sweet treats, maintaining elephants on hard substrates, and misusing disciplinary equipment were found to affect the health and welfare of the elephants. Comprehensive analyses of changes in elephant welfare with a historic perspective also emphasize that most mahouts now are inexperienced, with reduced qualifications for the growing use of elephants in tourism [10].

More recently, tigers were rescued from the illegal pet and hunting trades but then necessarily remained sheltered in facilities of various private organizations [11]. While interactions with tigers were initially passive in zoos (i.e., no-contact), recent industrial shifts led to increases in active tiger-tourist interactions (i.e., close contact, touching, feeding, taking photographs), especially with younger tigers. This shift led to creating jobs for tiger caregivers who are responsible for managing these interactions with tourists [2]. The human-animal relationship between caregivers and tigers is a modern development, more diverse, and significantly understudied, compared to that of the elephant-mahout relationship. Also, for both species, there is a lack of research on the caregivers’ perspectives of their relationships with their animals. Thus, an important first step for determining the associations between the tiger caregivers’ quality of life and their relationship with the tiger is to learn about the tiger tourism industry and the experiences of tiger caregivers.

The Thai government lacks adequate funding for oversight of domesticated elephants or wild tigers. While little is known about the tiger tourism facilities, most Thailand elephant sanctuaries do not meet the American standard of care. Furthermore, no social welfare assistance is available for animal caregivers. The impoverished mahouts and their families have been highly impacted; their limited income centers on the elephants. When elephants are sick or cannot work, the traditional mahouts and their families have no income. The classic tradition of one elephant per mahout involved a constant risk for food and housing insecurity and loss of livelihood. This precarious uncertainty had significant impacts on the mahouts’ psychological well-being, creating emotional trauma [12], poor quality of life, and jeopardized physical health for the mahouts and their families. To survive, traditional mahouts pushed elephants to their limits to feed both their families and the elephant. Social pressures experienced by the mahouts have similarly contributed to poor animal welfare all over the world [2,12].

To reduce the financial uncertainty arising from a one-elephant-per-mahout policy, sanctuaries in many countries, such as South Africa, Zimbabwe, Brazil, Sri Lanka, and India, have slowly shifted toward another management method where groups of elephants are managed by a team of keepers. This system can lead to some improvements in welfare for the humans and elephants involved [7,13,14,15] and has been implemented in some Thai elephant sanctuaries [14,15]. Additionally, various Thai organizations assist in managing elephant sanctuaries, such as the government agency for royal elephants that still follows a historic tradition, mahouts in small villages, private agencies at tourist attraction sites, and private agencies at sanctuaries or animal rehabilitation facilities [14]. Some of these are shifting toward protected contact, whereas others commonly still practice free contact. Collectively, the unique Thai historical context of elephants in Thai culture and the current situation changing uses of elephants throughout Thailand may influence more broadly the nature of relationships for mahouts and their elephants. Furthermore, differences in managing systems for Asian and African elephants may affect the quality and quantity of elephant-human interactions---both for the elephant and the human [16]. The rapid changes in mahouts’ jobs suggest their quality of life may be in jeopardy, both from having a less rewarding relationship with their elephant, and also because their salaries are insufficient and they lack other employee benefits, such as vacation and medical care.

Tiger tourism in Thailand is more recent, understudied, and less understood than elephant tourism. From 2001–2006, Prime Minister Thaksin Shinawatra shifted foreign animal exhibits, such as zoos or aquariums, to exotic animal interactive exhibits. These included hands-on contact with tigers [17], particularly with young tigers just a few years old. In a zoo setting, protected contact (e.g., tiger is separated from the human via a barrier) is the most common method to handle the tigers of varied ages; virtually all these tigers respond to commands from the trainer [18].

Based on Thailand’s Labor Protection Act, Thailand’s new minimum wages have been effective nationally since 1 January 2020. The minimum wages now range from 313 baht to 336 baht per day, equivalent to about $10 US [19]. A ‘day’ is defined as eight working hours in any 24 h or for work which may pose a threat to the health or safety of the employees, only seven working hours. The employers are forbidden to pay their employees less than the mandated rates.

Despite this new legislation on working hours, mahouts and tiger caregivers’ working conditions generally involve long hours and low salaries. Typical mahouts work 7–9 h per day and earn a monthly salary equal to about $50–$170 US per month, including food and housing benefits. Some facilities provide medical care for their elephants [1]. The tiger attraction sites provide human medical services whenever needed, but the facilities do not meet the minimal standard requirements for animal welfare [17].

Both elephant and tiger caregivers train and care for animals, but elephant and tiger caregivers are selected and trained in very different ways that likely affect their relationship with their animals. The two careers greatly differ in the amount of training they receive and the way by which they enter the career. Mahouts traditionally began their training in childhood and were informally taught by a family member, with boys often learning from their fathers [20,21]; even now, boys still may begin learning while very young. In contrast, new tiger trainers/caregivers spend 3–4 months in training before they can directly interact with tigers; typically, they have no family background and upbringing in tiger training.

Animal caregivers with favorable job characteristics can be assumed to have an overall higher quality of life than those with fewer benefits provided in their jobs. Those caregivers with more rewarding jobs perhaps would come to have a closer attachment and higher regard for their animal, shifting from a perspective of animal-as-worker towards a view of animal-as-companion or family member as the human’s quality of life improves. This outcome could improve the animal’s welfare if the human’s life improvement led to better animal treatment by the animal caregivers. While it is unclear how this type of tourism impacts the animal caregivers’ quality of life, differences in the sense of ownership, extent of time and contact with the animal, and organizational management styles are potential contributing factors. Some literature predicts that animal caregivers in multiple-caregiver management systems should have a better quality of life due to sharing of the effort [8,13,14,15]. However, it is difficult to separate the effects of the caregivers’ quality of life on the attachment to the animal from the effects of the management system or managing organization. For example, mahouts in one-to-one management systems may report lower quality of life judgments because of their financial uncertainty, yet they may be more likely to judge the animal-as-companion or family member and have better control of their elephants [20] due to a stronger personal connection to the specific elephant, in comparison to mahouts in multiple-caregiver management systems who have more financial stability but spend less time with any individual elephant.

Thai tiger caregivers have a different system of management than most elephant mahouts. For tigers, teams of caregivers share the care of all tigers, and no one has a one-to-one relationship with a tiger. Assessing a sample of mahouts and tiger caregivers from diverse backgrounds and contexts can help clarify the variables influencing both types of caregivers’ quality of life (e.g., job benefits, management systems, managing organizations) that may influence the caregiver’s attachment and caregiving of the animal.

This study explores the relationship between aspects of an animal caregiver’s quality of life and the relationship with the animal; specifically, are improvements in the quality of life on the job of the animal caregivers associated with improvements in the relationship with the animal, including how the person feels about the animal? This includes comparing the experiences of caregivers working in managing elephants versus those with tigers. Four potential influences on animal keepers’ quality of life and their relationships with their animals are addressed: (1) characteristics of caregivers, including their ages, personalized one-to-one vs. multiple-caregiver management systems, government vs. private facilities; (2) the effects on the relationship of closer, more extensive contact with the animal; (3) the caregivers’ perspectives on the essential requirements of excellent caregiving, and (4) the caregivers’ views of animal welfare in the tourism industry. While mahouts have been studied, this focuses specifically on their perspectives and to our knowledge this is the first systematic study of the tiger caregivers and the tiger tourism industry. We addressed the objectives outlined below, conducting in-depth, structured interviews in the Thai language with caregivers of elephants and tigers from multiple facilities and contrasting management systems.

### Objectives

1. Assess whether characteristics and experiences of elephant and tiger caregivers differ in government tourism facilities and private tourism facilities.
2. Compare the closeness of the relationship of animal caregivers having more extensive or long-term contact with their specific animals with caregivers having a shorter or less direct contact with animals.
3. Identify the essential qualities for excellent caregiving provided by elephant and tiger caregivers and whether they directly affect the animals and their welfare.
4. Describe the views of caregivers regarding the welfare of their animals working in tourism.

## 2. Materials and Methods

To assess the associations of caregiver quality of life with their views of their relationships with their animals, interviews of Thai-speaking mahouts and tiger caregivers were conducted at 4 private wildlife tourism facilities and 2 government zoos with contrasting management styles. Characteristics of the 6 sites are summarized in Table 1. Caregivers at each location granted interviews in person with audio-recording for 15–45 min in duration. Interviews were semi-structured with open-ended conversational questions and were parallel for elephant and tiger caregivers.

The total participants interviewed were 73 persons; 55 were elephant mahouts and 18 were tiger caregivers. The semi-structured interviews in the Thai language were recorded and information was subsequently transcribed in English, while also anonymizing the identity of interviewees. Each interviewee gave verbal consent and was offered the right to refuse to answer any questions and/or end the interview at any time. Interviews were conducted in Thai by a native Thai speaker (P.T.H.) to avoid language barriers and cultural biases. Interviewees were men working in the animal tourism industry at a facility managed either by government or by private companies in Thailand.

Quantitative data were gathered in interviews to assess economic and social aspects of the job, such as self-reported salary, number of hours worked, number of hours spent with family, and work satisfaction. To assess mahouts’ perceptions of their relationship with their elephant, mahouts categorized their relationships with their elephants as employees, friends, or family members.

Identical quantitative measures were collected across the elephant and tiger caregiver samples. Individualized quantitative measures on factors in this job affecting the quality of life and the relationship with the animal addressed lifestyle differences among elephant and tiger caregivers and their animals.

Several self-reported qualitative and quantitative measures of the jobs for elephant mahouts and tiger caregivers were tested. Chi square and Fisher exact tests were used.

## 3. Results

This research focuses on 4 major themes: mahouts’ and tiger caregivers’ characteristics of their jobs in their working environments; the closeness of their relationships with their animals; the essential qualities of animal caregivers; caregivers’ major concerns about the animals’ current welfare, including the benefits or problems resulting from the animal tourism industry.

### 3.1. Caregivers’ Characteristics

#### 3.1.1. Mahouts

**Figure 1:**
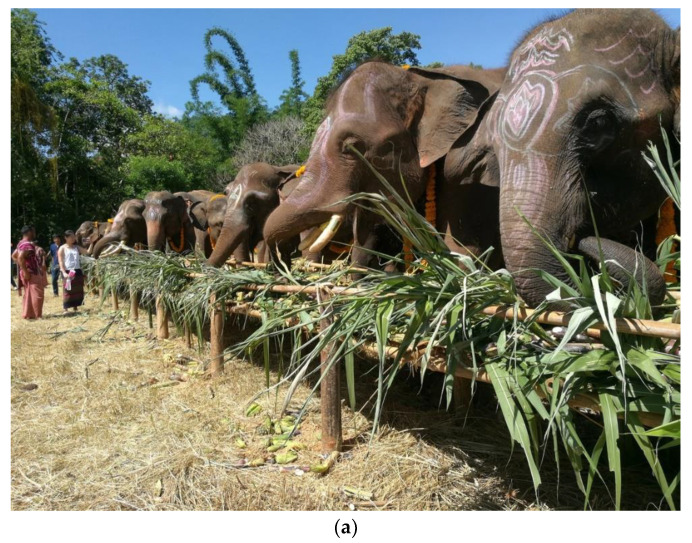

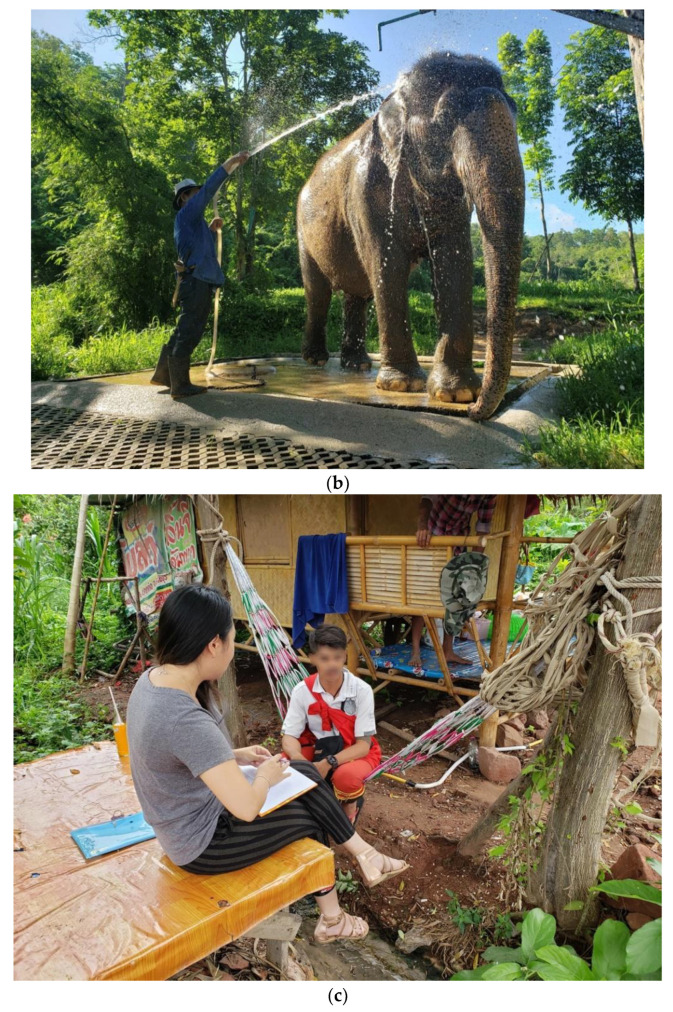
(**a**): A traditional annual ‘Shrine of Household God Worship’ ceremony. The elephants are offered sugarcane, banana, mango, and various types of food and worshiped by the local villagers. (**b**): Uncle ‘Ju’ (a senior mahout) is giving ‘Boon Nim’ a bath every morning after her breakfast. Boon-Nim is a retired geriatric elephant who has lost all her teeth. To prepare for her meal, Napier grass and sugarcane are ground up before mixing with feed pallets, tamarind paste, rock salt, and supplementals. (**c**): The interviewer was accepted at the workplace. A hut in the background is a house provided by the sanctuary.

Although involved in tourism, elephants and their mahouts in private tourist facilities maintain some of their traditional roles in the local community, including participation in festivals, as shown in Figure 1a. Most mahouts continue to have a one-to-one relationship with their own elephant and deliver personalized care, like this mahout caring for an elderly elephant, as in Figure 1b. Facilities welcomed the interviewer, even providing hospitality: Figure 1c.

Several substantial differences appeared between the mahouts working in private tourist facilities and those working in government facilities, including zoos. Those working in the private facilities were significantly younger than those working in government facilities (*p* < 0.0002), as shown in Table 2. Seven mahouts were less than 20 years old, and some working in tourism had become mahouts at 9–10 years of age. These mahouts had been recruited at very young ages; not wanting education, they had left their families. They live at the facility and work long hours, 5 a.m.–10 p.m. During the low season, they can go home for 1–2 months, and do some work at home if they have responsibilities. The private facilities paid significantly less than government (*p* = 0.0034) and provided less generous benefits for the mahouts and their families (*p* < 0.0001).

Private and government facilities also differed in the management style used with the elephants. Mahouts in private facilities were more likely to have a one-to-one relationship with the elephant than those in government facilities (*p* = 0.0217). Almost all mahouts in the private facilities had a one-to-one relationship with their elephant, whereas mahouts in government facilities were evenly split between one-to-one and group management of the elephants. All mahouts in private facilities had direct contact with their elephants, and almost all in government facilities; there was a statistical trend for more mahouts to use direct contact with the elephants in private facilities as compared with those in government zoos (*p* = 0.0513). Mahouts in both types of facilities almost universally assigned the elephants status equal to family members. Almost all mahouts in private facilities and all in government believed that the tourism industry brought some advantages for the animals.

Based on the 55 mahouts’ responses, the mean mahout’s salary was 11,308 baht/month, about $350 US. The mode salary was 9990 baht/month, about $300 US. In some facilities, the mahouts also earned tips from the tourists. Besides the salary and tips, full time mahouts at private facilities received benefits such as medical insurance, housing, and payment of utility bills. At the government facilities, full-time mahouts received similar benefits as the private facilities, plus a retirement fund and free tuition for their dependents. Part-time mahouts did not receive benefits.

Among these mahouts, 76% would recommend this job to their friends or relatives. Some of these mahouts would recommend the job specifically to a person who loves animals and would be dedicated to their job. Additionally, some of these mahouts would like to train their successor. The other 20% would not recommend it. Lastly, 4% did not answer.

#### 3.1.2. Tiger Caregivers

**Figure 2:**
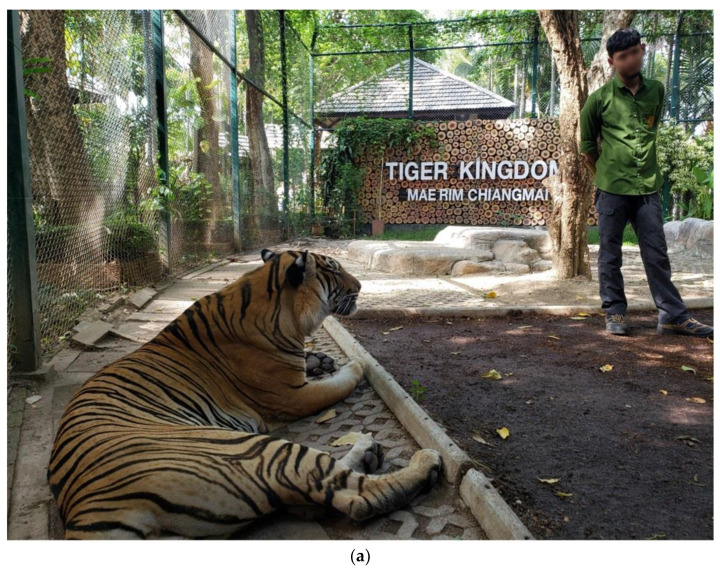

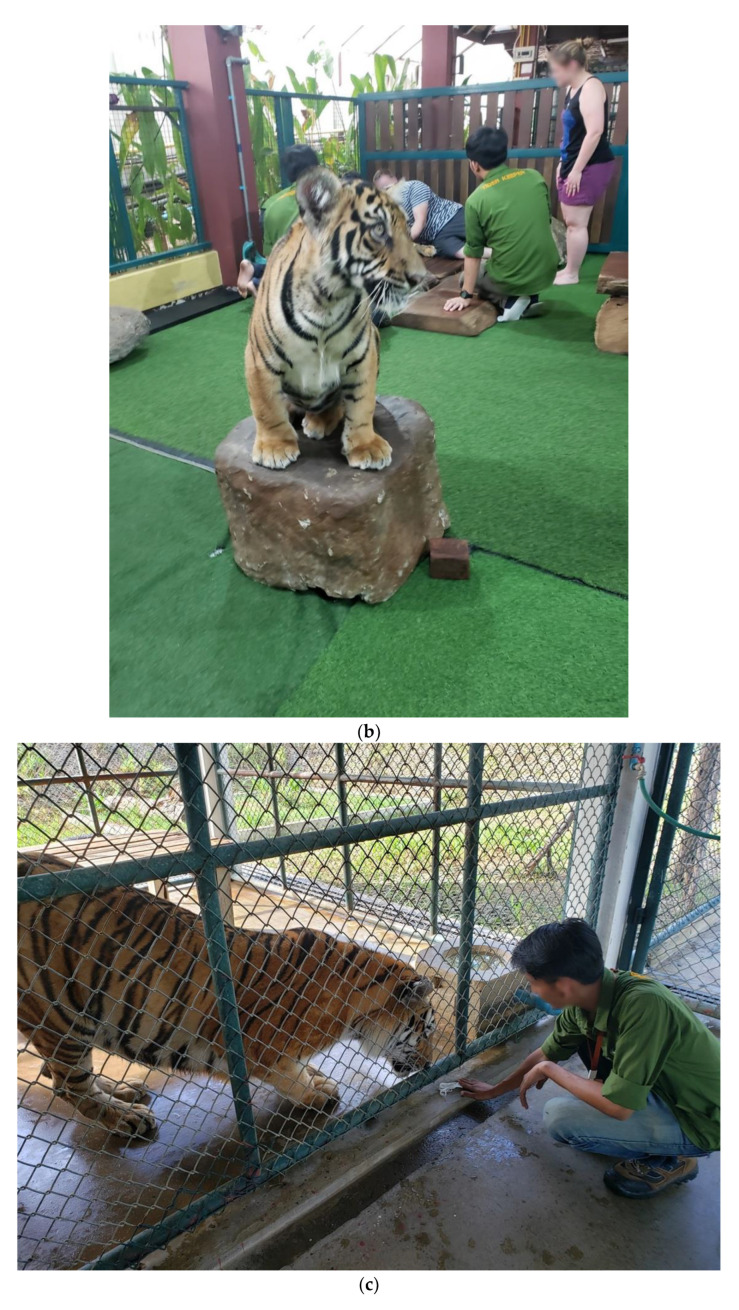

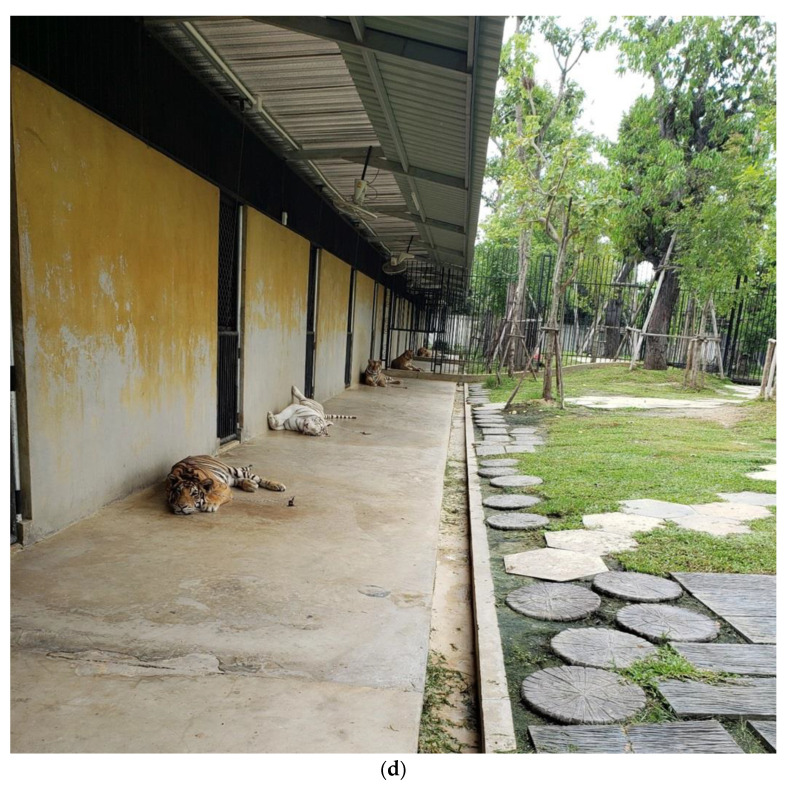
(**a**): An adult tiger and his caregiver are waiting for the tourists. The tiger is not chained and is able to move freely within the enclosure. This tourist enclosure has a small pool, toys, and varying terrain. The tiger’s caregiver is holding a foot-long bamboo stick (behind his back) which is a bridging stimulus used for training. (**b**): The tiger cubs are housed separately from the adult tigers. They are monitored with a security camera. The tourists can be in direct contact with the cubs under supervision of caregivers during the operational hours. Any cubs who express any stress behaviors would be returned and replaced with a new cub. (**c**): The caregiver offers a string toy to the retired tiger housed in an individual pen. Behind the pen is a common playground where the tiger has access to a pool, toys, hidden treats, and trees to encourage natural behaviors. Each retired tiger spends a few hours or more per day in the common playground. (**d**): The tigers are chained with an approximately three feet-long chains while waiting for the tourists and are housed in the pen adjacent to their chain posts.

While young tigers are typical in tourism, some adult tigers continue to meet tourists, as shown in Figure 2a. Primarily tiger cubs are introduced to tourists, with close supervision from the tigers’ caregivers, as in Figure 2b. Older tigers that have retired from meeting tourists can be given environmental enrichment, shown in Figure 2c. While waiting for meeting with tourists, tigers may be on short chains, as in Figure 2d.

Like the elephant mahouts, tiger caregivers in private facilities were significantly younger than those working in government facilities (*p* = 0.034), as shown in Table 2. While their salaries did not significantly differ, the private tourism facilities provided fewer benefits than government for the caregivers and their families (*p* = 0.004). A few of the tiger caregivers had salaries much higher than maximum salaries of mahouts, as shown in Table 3. Despite the disparities among salaries of mahouts and some tiger caregivers, salaries were not mentioned or highlighted as something they would change.

Tiger caregivers in private facilities all had direct contact with the young tigers provided for the tourists, whereas none of the caregivers in government facilities had direct contact with tigers: a highly significant difference despite the small number of tiger caregivers (*p* < 0.0001). Tiger caregivers in private facilities used group management; almost half of government facilities employed one-to-one management, but this was not a significant difference. Just over half of tiger caregivers working in private facilities and none in government facilities said that tourism brought benefits and specifically mentioned the animals as benefiting, a trend of a difference between the two types of facilities (*p* = 0.0539).

Based on the mean salary per month, the tiger caregivers earned more than the mahouts, 14,285 baht/month (about $440 US), but the mode salary of 10,000 baht/month was like that of mahouts. Full-time tiger caregivers at private facilities had benefits such as medical insurance and a retirement fund. Full-time tiger caregivers at the government facility received the same benefits as fulltime mahouts at the government facility, including medical insurance, housing, payment of utility bills, a retirement fund, and free tuition for their dependents. Like the mahouts, part-time tiger caretakers had no benefits beyond their salary.

Two facilities of this study that are owned by the government, Elephant Conservation Center and Khao Kheow Open Zoo, offer regular days off, averaging 1 day/week. The other facilities are owned by private companies and give day(s) off upon request. At the government facilities, the full-time tiger caregivers get 2 days off per week; part-time caregivers get 1 day off per week. At private tiger facilities, one facility offers 1 day off per week while the other facility offers a day off upon request.

Considering the tiger caregivers, 83% would recommend this job to their friends or relatives. Some of these tiger caregivers would only recommend this job if the person loves animals or the person has at least a high school diploma. The other 11% would not recommend this job. Lastly, 6% were indecisive.

### 3.2. Caregivers’ Relationships with Their Animals

As shown in Table 4, regardless of their ages, most mahouts considered their elephants as family members, and a lesser number referred to them as friends, rather than as just being employees. The tiger caregivers had an equal number viewing the tigers as family compared to viewing them as friends. Mahouts more often viewed their elephants as family members than did tiger caregivers with their tigers (*p* = 0.0318).

For the tiger caregivers, the specific management style, the working environment, and the caregivers’ ages were associated with their views. The caregivers who were older than 40 years typically worked for a government facility, while caregivers aged 20–40 years typically worked in tourism. As shown in Table 5, tiger caregivers working at the private tourism companies spent their time with the young tigers exclusively in a direct contact environment, as compared with those working at government facilities, who exclusively used protected contact.

### 3.3. Essential Qualities of Caregivers

The third theme focused on the most essential qualities of a good animal caregiver to learn whether the style of management or the caregivers’ ages would affect the list of the caregivers’ priorities. Caregivers each could designate the three qualities they viewed as most essential, reflecting their specific required job skills, their own priorities as an animal caregiver, and the quality of the job. Table 6 for the mahouts shows that 80% of the mahouts said ‘dedicated to care for the elephants’ as one of the three answers, 69% selected ‘love elephants’, and 22% selected ‘don’t punish the elephants without proper causes’. The main trends of the overall answers were love, dedication, and understanding the elephants, all focused on benefiting the elephants. These answers were more common than items such as, do not drink or smoke, or be an honest or nice person—personal attributes that perhaps seemed less pertinent for the job to the mahouts. When categorized by caregivers’ ages and types of management, their dedication to caring for the elephants and their love of elephants were still the main themes of the answers for each of the groups. The ages and the types of management did not affect the mahouts’ stated priorities for the important qualities needed to be a mahout.

Concerning the tiger caregivers, the overall trends of their answers resembled responses from mahouts. The most common theme was ‘love animals’ (67% gave as one of the three answers each could provide), followed by being calm (28%), being able to read a tiger’s body language and emotions (22%), and caring and considering the animal’s well-being (22%). The most common answers focused on two themes: love/care for the tigers’ well-being; and maintain a safe working environment for both humans and animals. When grouped by caregivers’ ages, the data showed similar results as the overall answers. When the data were grouped by types of management, some differences emerged between the private companies and the government agency or zoo management. At private companies, 92% had ‘love animals’ as one of the three essential qualities of a tiger caregiver. Regarding the secondary theme, 23% gave as one of the three answers: ‘care and considering animal well-being’ and ‘providing good service to tourists’. For tiger caregivers at the government agency, 60% had ‘calm’ as one of their three answers and 40% answered ‘brave’ as one of the three. ‘Love animal’ is the most common theme within the private facilities’ management where the tiger caregivers spend long hours working in direct contact sitting with the young animals. With zoo management, where the tiger caregivers spend short durations of interaction with the tigers, safety is the priority. ‘Calm’ is the most common theme at the zoos, with 60% of the caregivers answering as one of the three: ‘brave to work with the animals,’ answered by 40% of the caregivers.

### 3.4. Animal Welfare in the Tourism Industry

The fourth objective was to assess the animal’s caregivers’ thoughts about the current animal tourism industry and the future of the animals they care for.

#### 3.4.1. Caregivers’ Opinions on Their Animals Working

The 38 mahouts who viewed their elephants as family members were almost evenly split as to whether they agreed with using elephants in tourism, as shown in Table 7. The 6 caregivers of tigers viewing their animals as family members all supported tigers working in hands-on tourism, significantly differing from elephant mahouts, *p* = 0.0246. The tiger caregivers who viewed their tigers as family members liked them working, whereas those viewing them as employees disagreed with them working, a non-significant trend, *p* = 0.0657. Among caregivers who disagreed with their animals being used in tourism, most mahouts viewed their elephants as family members, whereas the tiger caregivers viewed their tigers as employees, *p* = 0.0107. One third of the tiger caregivers declined to answer the question regarding their tigers working; all six of these worked in tourism, and most of them viewed their tigers as friends.

Caregivers tended to prefer and support whichever management system they were working in. For example, among 4 mahouts working at the zoo, 3 disagreed with the act of elephants working in tourism and one who would have disagreed remained neutral due to the financial benefits the tourism provided.

Among the 6 mahouts working with group management systems, 2 strongly supported the tourism, one was neutral, and the other 3 disagreed with tourism, showing that those working in the group management style in the zoos were less supportive of using elephants in the tourism industry than those working in the private tourism facilities. Mahouts working in zoos preferred to have the elephants in a zoo environment with no direct contact between elephants and the tourists, sometimes mentioning they preferred that the elephants did not need to work. On the other hand, the mahouts whose work involved direct contact between elephants and tourists strongly agreed with using elephants in the tourism industry, believing that the elephants had a more natural lifestyle, sometimes being able to go into the forest where they could exercise natural behaviors. The mahouts who disagreed with using elephants to work preferred that the elephants roam freely in the forest fulltime like in the old tradition.

Among all tiger caregivers, 78% strongly agreed with using tigers in the tourist industry, regardless of the caregivers’ relationship with the tigers or working environment, especially if the animals were healthy, had not yet reached puberty, and were trained properly. The remaining 22% of caregivers disagreed with tiger tourism. More specifically, among the five caregivers working for the government at the zoo, 60% strongly agreed and 40% strongly disagreed on raising the tigers for work. But among tiger caregivers working for private tourism facilities, 46% refused to answer this question, and 38% strongly agreed on raising tigers for the tourism industry. These responses and refusals to answer this question suggest that tiger caregivers have considerable disagreement with use of tigers in tourism.

#### 3.4.2. Animal Welfare

Mahouts’ suggestions for improving animal welfare focused on improving the elephant living conditions and increasing the legal protections and regulations for elephants (Table 8). Tiger caregivers focused on improving the tiger’s quality of life and providing living conditions that allow the tigers to express their natural behaviors. Similar frequent responses from both the tiger and the elephant caregivers were that interviewees did not want to change anything. Among all interviewees, 25% wanted the animals to have access to larger areas where they can experience their natural habitats and express the range of their natural behaviors.

For the mahouts, the top answer, given by 40% of mahouts, was to make no changes. The next 4 most common answers focused on improving the elephant living conditions and improving the legal protections and regulations for elephants. The mahouts’ opinions showed that they believed the laws and registration of the domesticated elephant need to be updated for better protection of the elephants’ welfare.

The most frequent answer by tiger caregivers was to make no changes. The next 4 most common answers among tiger caregivers focused on improving the tigers’ quality of life and providing living conditions that allow the tigers to express their natural behaviors.

#### 3.4.3. Effects of Tourism for Animals and Caregivers

Animal caregivers may recognize some benefits of the wildlife tourism industry that affect the welfare of animals and humans. Responses for elephant mahouts and tiger caregivers are broken into two categories to compare both possible human benefits and animal benefits. Human benefits can include increased income for the caregivers, employment, education of staff or visitors, and tourist interactions. Animal benefits can include increased animal welfare or conservation.

Whether working in tourism or for government, almost all mahouts believed that tourism benefited the elephants (Table 1). However, only half of tiger caregivers working in tourism stated that the animals benefited, and none of the caregivers working for government.

## 4. Discussion

Suddenly ending Thailand’s huge logging industry resulted in many mahouts and their elephants who were without work, creating a unique situation for rapidly expanding the animal tourism industry. Now many elephants and their mahouts are employed, plus an increasing number of tigers with caregivers. The Thailand situation sharply contrasts with the more stable circumstance for elephants and mahouts in Nepal and India [20,21], where the numbers of elephants available for tourism have not been changing, nor have the number of mahouts’ jobs. Families that have worked as mahouts may often be continuing, with little need to recruit numerous new mahouts. Situated in the dynamic Thai setting, these interviews revealed the patterns of relationships and management of elephants and tigers, from the caregivers’ perspectives, in both private and government facilities.

### 4.1. Caregivers’ Characteristics

Thailand has gained economic benefits by employing elephants and tigers for tourism, adding new jobs with both elephants and tigers for young men who usually lack a family background of working with animals. While the traditional lifelong relationship of a mahout with the elephant in a family context is quickly disappearing in Thailand, the pattern of working with a single elephant and having direct contact persists. This means that a mahout develops a close relationship in which he knows his elephant very well. In recent decades, older mahouts have experienced the complex societal transitions of elephants, from logging, to street begging after the 1989 logging ban [7], and now to tourism [22]. Many older mahouts now work in government zoos where they have better benefits. In contrast, many younger mahouts now working in tourism, rather than learning about elephants from their fathers as formerly was traditional, leave home as young boys to take these jobs in tourism rather than attending school.

### 4.2. Caregivers’ Relationships with Their Animals

A relationship with an animal is fostered by the time together and working in partnership, providing opportunities for a possibility of building a close relationship. Mahouts, whether in tourism or government facilities, usually spend their time with a single elephant whom they get to know very well. Most of them consider their elephants as family members. In contrast with mahouts, tiger caregivers break into their jobs in tourism quickly and work with the changing array of young tigers at their private facilities. Thus, tiger caregivers in tourism are less likely to develop specific relationships with individual tigers. Fewer tiger caregivers considered their tigers as family members, and more of them proportionally considered their tigers as employees than with mahouts.

### 4.3. Essential Qualities of Caregivers

A strong majority of both mahouts and tiger caregivers emphasized the importance for their jobs of loving their animals, but even more mahouts mentioned that it is essential to be dedicated to caring for the elephants. Indeed, captive elephants require an extreme amount of care virtually 24 h a day. Elephants and tigers both are known to cause human fatalities, yet only a few caregivers of each species mentioned the importance of being brave. Tiger caregivers highlighted being calm as an important trait.

### 4.4. Animal Welfare in the Tourism Industry

Elephant tourism has addressed the problems of the mahouts being unable to provide food, medical care, and housing for the elephants, and the lack of available forest for them to freely roam. Mahouts could see these advantages. The situation had become life-threatening to the elephants and could have added to problems of illegal logging with poaching of lumber or elephants. Some mahouts disagreed with elephants working because they preferred a zoo environment where elephants would not have to work for food. Some preferred to raise an elephant in a forest like the old tradition where elephants can be close to nature and able to express natural behaviors. But Thailand’s large amount of forest land has been reduced from 60% of the total land area to 25% by 1998; after ending the logging and investing substantial effort, the forest land proportion subsequently had increased to 31.6% by 2016 [23]. Flooding and other disasters had made it essential for Thailand to end logging and limit elephants’ impact in forests. It became obvious that returning to the previous system where elephants freely exerted major impacts in the forests was no longer sustainable. Thus, for now it is prohibited for the domesticated elephants to roam in national forests.

Tiger caregivers perhaps faced a complicated context with their jobs working in tourism, knowing that their young tigers would quickly age out of their roles in tourism, even though some facilities may endeavor to provide well for older tigers. They saw minimal benefits for the animals and several declined to state whether they supported the use of tigers in tourism. Their concerns could potentially inform the preparation of guidelines to improve the animal tourism industry plus enhance the animals’ quality of life. The problems of breeding tigers to maintain a supply of tiger cubs for cub petting are well-known in the US, where more tigers live in captivity than the number in the wild worldwide [24].

Many of the mahouts and tiger caregivers, especially older ones, yearned to provide their animals with access to national forests or natural habitats. But the complex current situation the Thailand government faces involves sharply reduced forests, poaching of adult and young elephants, greater numbers of captive than wild elephants, diminished roles and status for mahouts, and inadequate forests to support the captive elephants [25]. The contentious social terrain yields no perfect solution. This survey of elephant mahouts and tiger caregivers provides views of their experiences.

### 4.5. Limitations

The project’s limitations are that the interviews were conducted at several facilities that were welcoming and supportive of conducting interviews with mahouts and/or tiger caregivers. These facilities aspire to enhance the well-being for their caregivers and animals. The responses of the caregivers thus do not necessarily represent an average throughout Thailand of caregivers’ perspectives. Some caregivers may have felt uncomfortable being candid with their concerns regarding their animals’ welfare, particularly the tiger caregivers who declined commenting on whether they supported use of tigers in tourism.

## 5. Conclusions

Government management at the zoos in Thailand is starting to reduce direct contact with elephants and is shifting toward group management rather than one-to-one relationships; this somewhat limits the development of a close relationship of man and mahout, compared to the one-to-one direct contact of private tourism. Yet, both systems provide a better and more stable quality of life for the animals and the mahouts compared to the period prior to 1989. These interviews reveal that the elephant tourism industry is providing some quality of life with basic needs for elephants and their mahouts, and tigers and their caregivers. This animal tourism is providing perhaps only a temporary and imperfect solution to the challenges. A central regulatory body to uphold the standard of care for the animals used in the tourism industry would be useful to advocate for quality of life for these animals and their caregivers.

## Figures and Tables

**Table 1:** Characteristics of tourism facilities, animal species, and styles of animal management.

	Locations	Management	Animals	Number Interviewed and Management Style
1.	Chang Puak Camp Damnoensaduak, Ratchaburi (middle region)	Private company; direct contact between elephants and tourists; elephant talent shows, riding and bathing with elephants; tiger cubs petting zoo, photos with tigers	~30 elephants, ~20 tigers, monkeys, etc.	9 mahouts; one-to-one relationship, direct contact and 4 tiger caregivers; multiple caregivers for animal group, direct contact.
2.	Thai Elephant Conservation Center, Lumpang (northern region)	Government agency: royal elephants; free elephant hospital, offer elephant and mahout training classes, direct contact between; riding and bathing with elephants; elephant shows; home stay	~48 elephants	25 mahouts; one-to-one relationship, direct contact
3.	Khao Kheow Open Zoo, Chonburi (middle region)	Government agency, in house veterinary hospital, indirect contact between elephants and tourists; elephant feeding behind a fence; no riding; no direct contact with tigers, but observation	~8 elephants, ~20 tigers, wild cats, etc.	4 mahouts; multiple caregivers for animal group, direct contact and 5 tiger caregivers: multiple caregivers for animal group, protected contact
4.	Into the Wild Elephant Camp, Chiang Mai	Private company, direct contact between elephants and tourists; bathing with elephant; viewing elephant in habitat; no riding	~15 elephants	2 mahouts; multiple caregivers for animal group, direct contact
5.	Patara Elephant Farm, Chiang Mai	Private company, in house elephant hospital, direct contact between elephants and tourists	~150 elephants	16 mahouts; one-to-one relationship, direct contact
6.	Tiger Kingdom, Chiang Mai	Private company; in- house tiger hospital; direct contact between tigers and tourists; petting tiger cubs, photos with tigers, tiger shows	~200 tigers	9 tiger caregivers; multiple caregivers of animal group, direct contact

**Table 2:** Characteristics and perspectives of caregivers.

**Mahout’s Characteristics**	**Characteristic Categories**	**Private Facility** ***n* = 26** **Mahouts (%)**	**Government Facility** ***n* = 29** **Mahouts (%)**	***p*-Value**
Age	≤40 years	23 (85.19%)	10 (35.71%)	<0.0002
	>40 years	4 (14.81%)	18 (64.29%)	
Income	<฿10 k/month	15 (62%)	9 (37.50%)	=0.0034
	≥฿10 k/month	5 (19.23%)	21 (80.77%)	
	Declined	2	2	
	Missing	0	1	
Benefits	No Benefits	1 (4%)	3 (11.54%)	<0.0001
	Yes, employee only	24 (96%)	0 (0%)	
	Yes, employee/family	0 (0%)	23 (88.46%)	
	Missing	1	3	
Direct Contact with Animals	No	0 (0%)	4 (13.79%)	=0.0513
	Yes	26 (100%)	25 (86.21%)	
Relationship	Gives elephant status equivalent to human relationship	24 (92.31%)	28 (96.55%)	
Management	One-to-one relationship	20 (76.92%)	14 (50%)	<0.0287
	Group management	6 (23.08%)	14 (50%)	
Tourism Industry Brings Benefits	Animals mentioned	21 (87.5%)	29 (100%)	<0.0864
	No animal mention	3 (12.50%)	0	
**Tiger Caregivers’** **Characteristics**	**Characteristic Categories**	**Private Facility** ***n* = 13** **Caregivers (%)**	**Government Facility** ***n* = 5** **Caregivers (%)**	***p*-Value**
Age	≤40 years	10 (76.92%)	1 (20.00%)	=0.034
	>40 years	3 (23.08%)	4 (80.00%)	
Income	<฿10 k/month	7 (53.85%)	3 (60%)	=0.36
	≥฿10 k/month	2 (15.38%)	2 (40%)	
	Declined	4	0	
Benefits	No Benefits	0 (0%)	2 (40.00%)	=0.004
	Yes, employee only	9 (69.23%)	0 (0%)	
	Yes, employee/family	4 (30.77%)	3 (60%)	
Direct Contact with Animals	Yes	13 (100%)	0 (0%)	<0.0001
	No	0 (0%)	5 (100%)	
Relationship	Gives tiger status equivalent to human relationship	5 (38.46%)	2 (40.00%)	0.4444
Management	One-to-one relationship	1 (7.9%)	2 (40%)	=0.1593
	Group management	12 (92.31%)	3 (60%)	
Tourism Industry Brings Benefits	Animals mentioned	7 (53.85%)	0	<0.0539
	No animal mention	6 (46.15%)	5 (100%)	

**Table 3:** Salaries reported by mahouts and tiger caregivers.

Salaries	Mahouts *n* = 55 (%)	Tiger Caregivers *n* = 18 (%)
฿300–333 /day < or equal ฿12,000/month	34 (62%)	9 (50%)
฿400/day < or equal ฿15,000/month	7 (13%)	1 (6%)
฿500/day < or equal ฿18,000/month	6 (11%)	1 (6%)
฿600/day	3 (5%)	1 (6%)
฿1000/day	--	2 (11%)
Not answering	5 (9%)	4 (22%)

**Table 4:** Relationship with the animal reported by mahouts and tiger caregivers, as related to caregiver’s age.

Caregiver Types	Age Categories	Family Member	Friend	Employee
Elephant Mahouts	≤40 years	22	9	2
	>40 years	16	5	1
	Total * (% of all)	38 (69.09%)	14 (25.45%)	3 (5.45%)
Tiger Caregivers	≤40 years	3	6	2
	>40 years	4	1	2
	Total * (% of all)	7 (38.89%)	7 (38.89%)	4 (22.22%)

* Elephant mahouts as a group significantly differ from tiger caregivers as a group in their relationships with their animals: 2 × 3 Fisher’s test: *p* = 0.0318.

**Table 5:** Caregiver’s relationship with the animal and facility context, with direct contact versus protected contact.

Caregiver Types	Perceived Relationship	Direct Contact	Protected Contact
Elephant Mahout-Relationship	Family member	35	3
	Friend	13	1
	Employee	3	0
Tiger Caregiver Relationship	Family member	5	2
	Friend	6	1
	Employee	1	2
Elephant Mahout Facility	Private	27	0
	Government	24	4
Tiger Caregiver Facility	Private	13	0
	Government	0	5

**Table 6:** Essential qualities for animal caregivers (among three choices, each provided by at least two caregivers).

**Mahouts’ Responses**	**Number of Mahouts: *n* = 55**
Be dedicated to care for the elephants	44 (80%)
Love elephants	38 (69%)
Do not scold the elephants without proper causes	12 (22%)
Be diligent: offer Napier grass, take them for a walk and bath, etc.	10 (18%)
Be disciplined: provide feed, medication, routine cares on time, etc.	9 (15%)
Be able to read an elephant’s body language and emotions	9 (15%)
Gentle/ be kind to an elephant	5 (9%)
Have an experience of working with elephants	4 (7%)
Be brave to work with the elephants	3 (5%)
**Tiger Caregivers’ Responses**	**Number of Caregivers: *n* = 18**
Love animals	12 (67%)
Be calm	5 (28%)
Be able to read a tiger’s body language and emotions	4 (22%)
Care and consider the animal’s well-being	4 (22%)
Be brave	3 (17%)
Be service-minded	3 (17%)
Be disciplined; provide feed, medication, routine cares on time etc.	3 (17%)
Love the job	2 (11%)
Be a team player	2 (11%)

**Table 7:** Caregivers’ perspectives on animal tourism.

**Mahouts**	**Perceived Relationship**	**Agree with Animal in Tourism** ***n* = 22**	**Disagree with Animal in Tourism #** ***n* = 31**	**Decline to Answer** ***n* = 2**
How mahouts viewed the elephant	Family member *	18 (81.82%)	20 (64.52%)	
	Friend	3 (13.64%)	9 (29.03%)	2
	Employee	1 (4.55%)	2 (6.45%)	
**Tiger Caregivers**	**Perceived Relationship**	**Agree with Animal Working with Hands-On Tourist Interactions** ***n* = 9**	**Disagree with Animal Working with Hands-on Tourist Interactions #** ***n* = 3**	**Decline to Answer** **(All at Private Facilities)** ***n* = 6**
How the caregiver viewed the tiger +	Family member *	6 (66.67%)	0 (0%)	1
	Friend	2 (22.22%)	1 (33.33%)	4
	Employee	1 (11.11%)	2 (66.67%)	1

* Caregivers of elephants and tigers who viewed their animals as family members significantly differed regarding their animals working in tourism: Fisher’s test, *p* = 0.0246. + Caregivers of tigers who agreed or disagreed with tigers working in tourism showed a trend to differ in whether they viewed the animal as a family member, friend, or employee: Chi square 8.8214, 4 df, *p* = 0.0657. # Caregivers of elephants and tigers who disagreed with their animals working in tourism significantly differed regarding whether they viewed the animal as a family member, friend, or employee: 2 × 3 Fisher’s test, *p* = 0.0107.

**Table 8:** Caregivers’ responses for suggested improvements in animal welfare (unlimited choices; each response provided by at least 10% of caregivers).

**Mahouts’ Responses**	**Number of Mahouts: *n* = 55 (%)**
Does not want to change anything	22 (40%)
Allow elephants in the national forests if they do not create conflict with the local farmers	13 (24%)
End the inhumane elephant performances, torturing while training, illegal logging	9 (16%)
More support from the government: improve laws and regulations, conserve Asian elephants, muster a task force, create long-term plans for the elephant’s well-being	9 (16%)
Improve elephant’s quality of life: provide proper animal husbandry, provide veterinary services with a rigid report system and punishment for the mistreatment of elephants	8 (15%)
Do not put elephants to work	6 (11%)
**Tiger Caregivers’ Responses**	**Number of Caregivers: *n* = 18 (%)**
Does not want to change anything	5 (18%)
Wants larger habitats, an opened zoo	5 (18%)
Suggests attracting more tourists: improve the layout of the habitats, increase limited breeding of tigers, add interactions between tourist and the tiger’s cubs	3 (11%)
Initiate employees’ right to bargain with the operation for the tiger’s best interest; improve employee feedback system	3 (11%)

## Data Availability

The surveys and data presented in this study are openly available in Figshare at 10.6084/m9.figshare.19337795.
